# Thalassemias: can we reduce the national burden?

**DOI:** 10.1186/1755-8166-7-S1-I43

**Published:** 2014-01-21

**Authors:** Roshan Colah

**Affiliations:** 1 ICMR-National Institute of Immunohaematology, Parel, Mumbai, India

## 

The burden of inherited disorders of hemoglobin, the commonest group of single gene disorders in India is huge. With a population of 1.21 billion and an average prevalence of β-thalassemia carriers being around 3.5-4%, there would be 35-45 million carriers and the estimated number of births of affected babies annually would be 10,000-12,000. The carrier rates vary from 1-17% in different ethnic groups. Apart from β-thalassemia, Hb E is common in the north eastern region and in West Bengal (4 to > 50%) and Hb S is prevalent in parts of central, western and eastern India (5-40%). Thus interaction of the β-thalassemias with these Hb variants is not uncommon and can lead to a severe disorder.

One way to combat the burden is by prenatal diagnosis but the only approach to reduce the national burden is by a comprehensive community control programme. Awareness is very limited in different states (<20% among pregnant women) and the entire public health infrastructure from medical colleges to district hospitals and down to the community health centres must be mobilized for education and generating awareness on these disorders. Experience shows that screening for carriers in India will have to be done at multiple levels – schools, colleges, antenatal clinics as well as cascade screening where extended family members of an affected child are screened. However, antenatal screening with subsequent testing of the husbands of carrier women would be the most cost effective way to identify couples at-risk and give them the option of prenatal diagnosis. For this, obstetricians must recognize the implications of hypochronic and microcytic red cell indices (MCV <80 fl, MCH < 27 pg and a high RBC count) and ask for a β-thalassemia screen by estimation of HbA_2_ levels. Several laboratories in the country use automated HPLC for reliable HbA_2_ estimation and identification of heterozygotes is not a problem. Late registration at antenatal clinics (only 15-20% in the first trimester in public hospitals) is an impediment resulting in many couples at-risk being identified late and requiring second trimester fetal diagnosis. Social stigmatization is an issue to be dealt with during premarital screening of marriage partners of carrier individuals. Only education can reduce this barrier. Many State Governments in India are now undertaking population screening and counselling programmes and Gujarat and West Bengal have taken the lead.

There are 10-12 centres offering prenatal diagnosis by CVS and DNA analysis and recently the Indian Council of Medical Research has established 6 more centres in different regions. However, many more centres would be required once there is an increasing demand. The spectrum of mutations and their distribution are now known which would facilitate prenatal diagnosis.

Thus, there are many challenges – a large and diverse population, limited awareness, late registration in antenatal clinics and inequality of available services (urban v/s rural areas) with around 70% of the population residing in rural areas. There is a need for the Central and State Governments to join hands and involve NGO groups to form networks in different regions which when backed by political will could gradually reduce the national burden of hemoglobinopathies in this vast country.

